# A Holistic Approach to Analyze Systemic Jasmonate Accumulation in Individual Leaves of *Arabidopsis* Rosettes Upon Wounding

**DOI:** 10.3389/fpls.2018.01569

**Published:** 2018-10-30

**Authors:** Monika Heyer, Michael Reichelt, Axel Mithöfer

**Affiliations:** ^1^Department of Bioorganic Chemistry, Max Planck Institute for Chemical Ecology, Jena, Germany; ^2^Department of Biochemistry, Max Planck Institute for Chemical Ecology, Jena, Germany

**Keywords:** systemic signaling, γ-aminobutyric acid, wounding, MecWorm, plant defense, phytohormones, jasmonates, *JASMONATE-ZIM DOMAIN (JAZ) 10*

## Abstract

Phytohormones, especially jasmonates, are known to be mediators of the plant responses to wounding and herbivore feeding. Their role in such stress responses has been largely studied locally in treated leaves. However, less is known about the induced systemic distribution of phytohormone signals upon these kinds of stresses. Here, a holistic approach was performed in order to investigate the systemic phytohormone pattern in the rosette of *Arabidopsis* *thaliana* after herbivore-related wounding. Levels of different stress-related phytohormones such as jasmonates, abscisic acid, and salicylic acid were analyzed in individual leaves. We demonstrate that the typically used sampling method, where leaves are first cut and immediately frozen, causes false-positive results since cutting already induces systemic jasmonate elevations within less than 1.6 min. Therefore, this approach is not suitable to study systemic phytohormone changes in the whole plant. By developing a new method where leaves are frozen first and subsequently cut, sampling-induced phytohormone elevations could be reduced. Using this new method, we show that jasmonic acid and its active isoleucine conjugate (jasmonoyl-isoleucine) are involved in the fast systemic wound response of *Arabidopsis*. A systemic induction of the jasmonates’ precursor, 12-oxo-phytodienoic acid, was not observed throughout our treatments. The systemic phytohormone distribution pattern is strongly linked to the vascular connections between the leaves, providing further evidence that the vascular system is used for long distance-signaling in *Arabidopsis*. Besides already known vascular connections, we also demonstrate that the systemic distribution of jasmonate signals can be extended to distant leaves, which are systemically but indirectly connected *via* another vascularly connected leaf. This holistic approach covering almost the whole *Arabidopsis* rosette introduces a method to overcome false-positive results in systemic phytohormone determinations and demonstrates that wounding-induced long-distance signaling includes fast changes in jasmonate levels in systemic, non-treated leaves.

## Introduction

In order to survive insect attacks, plants developed a variety of defense strategies that are locally induced by feeding of the herbivore (Mithöfer and Boland, 2012). Since the insect can easily move to other parts of the plant, it is essential for the plant to defend the non-fed systemic leaves as well as the local fed ones. Several studies have shown that defensive compounds accumulate both locally and systemically, like proteinase inhibitors in potatoes, tannins in aspen or γ-aminobutyric acid (GABA) in *Arabidopsis thaliana* (Pena-Cortes et al., 1988; Peters and Constabel, 2002; Scholz et al., 2015, 2017). To ensure a fast reaction to the insect feeding, these defensive compounds are most probably produced directly in the systemic parts rather than being transported from local to systemic leaves (Peters and Constabel, 2002; Scholz et al., 2017). This implies that there are signals traveling throughout the whole plant inducing systemic defenses. Several signals have been discussed to mediate systemic reactions to herbivores or herbivore-related stimuli, such as hydraulic changes (Farmer et al., 2014), electric signals (Mousavi et al., 2013; Salvador-Recatalà et al., 2014; Zimmermann et al., 2016), calcium waves (Kiep et al., 2015), reactive oxygen species (Miller et al., 2009), and phytohormones (Glauser et al., 2008a, 2009; Sato et al., 2009, 2011; Vos et al., 2013; Gasperini et al., 2015; Jimenez-Aleman et al., 2015).

Lacking a nervous system for rapid long distance signal transport, plants use their vascular system to transfer those systemic signals (Farmer et al., 2014; Salvador-Recatalà et al., 2014; Gasperini et al., 2015; Kiep et al., 2015; Nguyen et al., 2018). In *Arabidopsis*, leaves are connected *via* so called parastichies in a specific pattern according to their development: leaf n is directly connected to leaves n ± 5 and n ± 8 (connections of first order) and indirectly to leaves n ± 3 (connections of second order) (Dengler, 2006; Mousavi et al., 2013) (see example for leaf 8 parastichies Supplementary Figure S1). It was shown for several of the above mentioned systemic signals that their distribution among the *Arabidopsis* rosette corresponds to the vascular connections between the leaves, such as for leaf surface potential changes (Mousavi et al., 2013; Nguyen et al., 2018) or calcium signals (Kiep et al., 2015; Nguyen et al., 2018). Also for the phytohormone jasmonic acid (JA) and its active isoleucine conjugate (jasmonoyl-isoleucine, JA-Ile) that are known to be key regulators of the plant response to herbivory (Howe and Jander, 2008), there are hints that their systemic pattern corresponds to the parastichies between leaves. It was shown that the expression pattern of the JA responsive gene *JAMONATE ZIM-DOMAIN10* (*JAZ10*) among the whole *Arabidopsis* rosette follows the parastichious connections of the treated leaf (Mousavi et al., 2013). Furthermore Glauser et al. (2009) published that if two parastichious connected leaves are both wounded, a third non-treated parastichious connected leaf shows an increase in JA, but not an unconnected leaf. Similar results were obtained for JA-Ile (Chauvin et al., 2013) and a precursor of JA and JA-Ile, 8-oxopentenylcyclopentenyloctanoic acid (OPC-8:0) (Jimenez-Aleman et al., 2015).

Strikingly, a holistic study instead of picking selected leaves to investigate the phytohormone level among the whole *Arabidopsis* rosette and by this linking the phytohormone pattern to the known parastichies, is still missing. Even though a lot of work has been done on systemic jasmonates, less is known about other phytohormones. However, it has been long time speculated that salicylic acid (SA) as antagonist of JA might play a role in herbivore defense as well (Koornneef and Pieterse, 2008). Additionally ABA is known to regulate herbivore defense and to play a role in priming of systemic leaves (Bodenhausen and Reymond, 2007; Vos et al., 2013).

Here we present data, unraveling the systemic pattern of jasmonates as well as SA and ABA among the whole *Arabidopsis* rosette, by directly quantifying individual leaf phytohormone levels. We demonstrate that the sampling method is crucial for investigating the holistic jasmonate pattern. Furthermore, our data suggest that mainly JA and JA-Ile rather than their biosynthetic precursor 12-oxo-phytodienoic acid (OPDA) or SA and ABA mediate fast systemic herbivory-related defense responses.

## Materials and Methods

### Plant Growth, Treatment and Sampling Procedures

**FIGURE 1:**
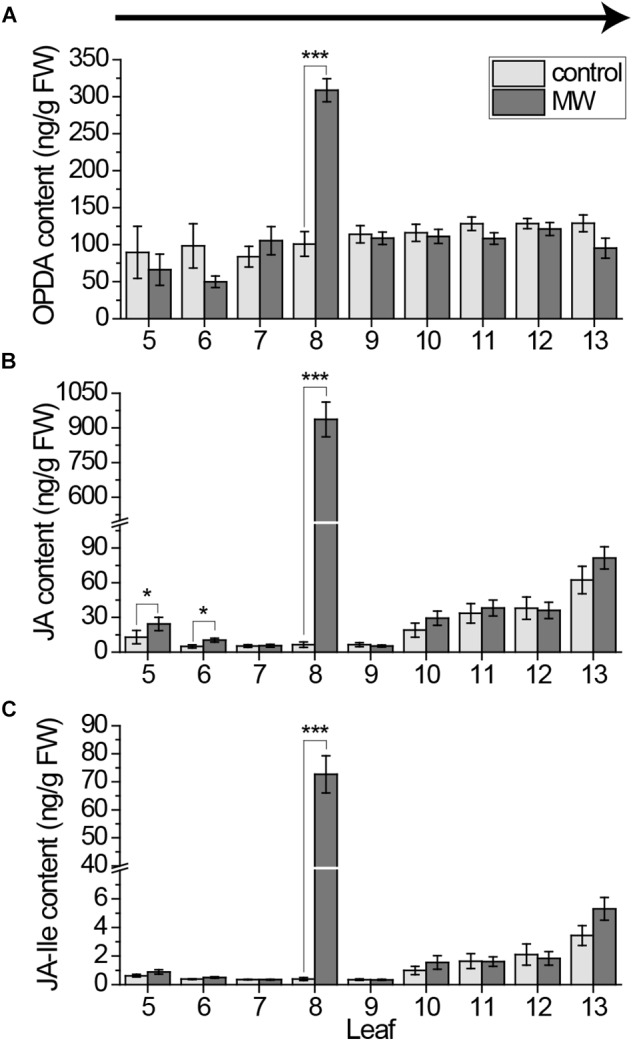
Jasmonate contents in individual *Arabidopsis* leaves after wounding, sampled with the cut-and-freeze-method from old to young leaves. Mean levels of OPDA **(A)**, JA **(B)**, and JA-Ile **(C)** ±standard error (SE) in individual leaves after wounding leaf 8 with MecWorm for 1 h. Untreated plants were used as control. Legend for color code see **(A)**. Leaves were collected with the cut-and-freeze-method from leaf 5 (old) to leaf 13 (young) as indicated by the arrow in **(A)**. The experiment was repeated four times independently (*n* ≥ 15). Statistically significant differences between phytohormone content of treated and untreated plants within the leaves were determined by unpaired two-sample Wilcoxon test. Asterisk indicates significance (^∗^*P* < 0.05, ^∗∗∗^*P* < 0.001). *P*-values are False discovery rate (FDR) corrected.

**FIGURE 2:**
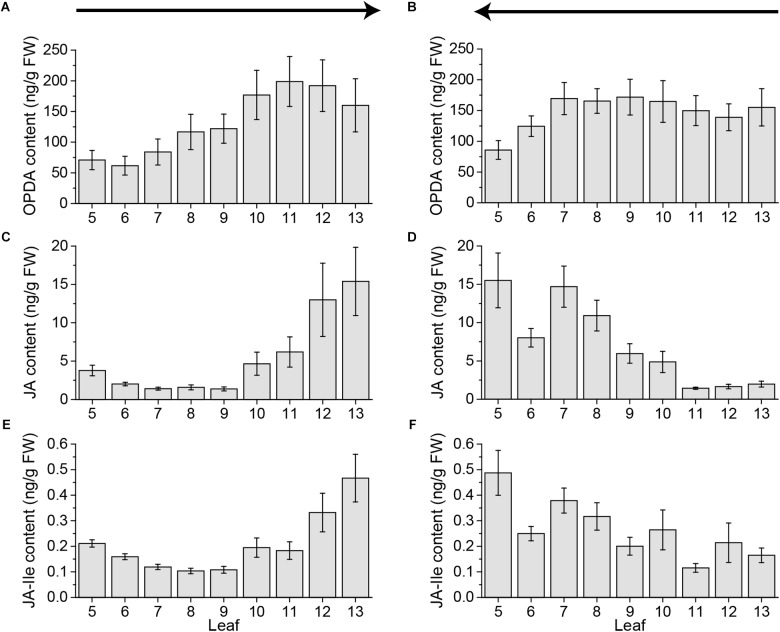
Jasmonate contents of untreated *Arabidopsis* leaves. Mean levels of OPDA **(A,B)**, JA **(C,D)**, and JA-Ile **(E,F)** ± SE in individual leaves of untreated *Arabidopsis* plants. Single leaves were collected with the cut-and-freeze-method from leaf 5 (old) to leaf 13 (young) **(A,C,E)** or from young to old **(B,D,F)**. Sampling directions are indicated by arrows. The experiment was repeated two times independently (*n* ≥ 11).

Five week old *A. thaliana* Columbia-0 plants were used for all experiments. Plants were grown as described before (Scholz et al., 2017). Leaves were counted prior the treatment according to Farmer et al. (2013); the younger the higher the number. Plants were mechanically wounded at leaf 8 using MecWorm (Mithöfer et al., 2005) to ensure a continuous wounding. For 1 h treatment two rectangular shaped areas, each with a duration of 30 min, using a speed of 12 punches per minute, were designed to wound leaf 8 (Scholz et al., 2017). The midrib of the leaf was included in wounding. After treatment all leaves in the range of leaf 5 to leaf 13 were collected either from young to old or from old to young leaves. The sampling direction is mentioned in the respective figure legends. For experiments presented in Figures 1, 2, 4, 5, 6, 9 one leaf after another was cut and directly frozen following the sampling method of Glauser et al. (2008b) (further referred to as cut-and-freeze-method). For other experiments the freeze-and-cut-method was developed. The whole rosette was dipped in liquid nitrogen for 5–10 s. Leaves were cut of the frozen rosette starting from young leaves to old ones. Immediately after cutting each leaf was kept in liquid nitrogen. For all extraction procedures leaf material was ground in precooled cryoblocks using 2010 Geno/Grinder^®^ (SPEX^®^SamplePrep, Metuchen, NJ, United States). Samples were stored in -80°C till the extraction procedure.

### Extraction and Quantification of Phytohormones

Whole leaf was used for phytohormone extraction and weighed in frozen for quantification of the metabolites. Phytohormones were extracted following the single leaf extraction protocol described in Jimenez-Aleman et al. (2015) with some modifications. For phytohormone measurements shown in Figures 1, 5E,G samples were extracted using 1.0 ml methanol containing 40 ng D_6_-ABA (Santa Cruz Biotechnology, Santa Cruz, CA, United States), 40 ng of D_6_-JA (HPC Standards GmbH, Cunnersdorf, Germany), 40 ng D_4_-SA (Sigma-Aldrich) and 8 ng of JA-^13^C_6_-Ile conjugate as internal standard. All other extractions (Figures 2, 4, 5A–D,F,H, 7, 8) were performed using D_6_-JA (HPC Standards GmbH, Cunnersdorf, Germany), D_4_-SA (Santa Cruz Biotechnology, Santa Cruz, CA, United States), D_6-_ABA (Toronto Research Chemicals, Toronto, ON, Canada), D_6_-JA-Ile (HPC Standards GmbH, Cunnersdorf, Germany) as internal standards, respectively. Chromatographic separation of phytohormones was performed on an Agilent 1260 HPLC system (Agilent Technologies, Santa Clara, CA, United States) using a Zorbax Eclipse XDB-C18 column (50 × 4.6 mm, 1.8 μm, Agilent). Formic acid (0.05%) in water and acetonitrile were employed as mobile phases A and B. The elution profile was: 0–0.5 min, 10% B; 0.5–4.0 min, 10–90% B; 4.0–4.02 min, 90–100% B; 4.02–4.50min, 100% B, 4.50–4.51 min 100–10% B and 4.51–7.00, 10% B. The mobile phase flow rate was 1.1 ml/min and column temperature was maintained at 25°C. Phytohormones were determined using an API 5000 tandem mass spectrometer (Applied Biosystems, Foster City, CA, United States) with a turbospray ion source operated in negative ionization mode. The instrument parameters were optimized by infusion experiments with pure standards, where available. The ion spray voltage was maintained at -4500 eV. The turbo gas temperature was set at 700°C. Nebulizing gas was set at 60 psi, curtain gas at 25 psi, the heating gas at 60 psi and collision gas at 7 psi. Multiple reaction monitoring (MRM) was used to monitor analyte parent ion → product ion fragmentations as follows: m/z 136.9 →93.0 [collision energy (CE) -22 V; declustering potential (DP) -35 V) for SA; m/z 140.9 →97.0 (CE -22 V; DP -35 V) for D_4_-SA; m/z 290.9 →165.1 (CE -24 V; DP -45 V) for OPDA; m/z 209.1 →59.0 (CE -24 V; DP -35 V) for JA; m/z 215.1 →59.0 (CE -24 V; DP -35 V) for D_6_-JA; m/z 214.1 →59.0 (CE -24 V; DP -35 V) for D_5_-JA; m/z 322.2 →130.1 (CE -30 V; DP -50 V) JA-Ile; *m/z* 328.2 →136.1 (CE -30 V; DP -50 V) for JA-^13^C_6_-Ile; m/z 328.2 →130.1 (CE -30 V; DP -50 V) for D_6_-JA-Ile; m/z 327.2 →130.1 (CE -30 V; DP -50 V) for D_5_-JA-Ile; m/z 263.0 →153.2 (CE -22 V; DP -35 V) for ABA; m/z 269.0 →159.2 (CE -22 V; DP -35 V)] for D_6_-ABA. Both Q1 and Q3 quadrupoles were maintained at unit resolution. Analyst 1.6 software (Applied Biosystems) was used for data acquisition and processing. Linearity in ionization efficiencies was verified by analyzing dilution series of standard mixtures. Phytohormones were quantified relative to the signal of their corresponding internal standard. Since it was observed that both the D_6_-labeled JA and D_6_-labeled JA-Ile standards (HPC Standards GmbH, Cunnersdorf, Germany) contained 40% of the corresponding D_5_-labeled compounds, the sum of the peak areas of D_5_- and D_6_-compound was used for quantification. For quantification of OPDA the internal D_5_-/D_6_-JA standard was used applying experimental-determined response factors of 0.5, respectively. The response factors were determined by analyzing a mixture of OPDA [kindly provided by W. Boland, MPI for Chemical Ecology, Jena, Germany; synthesized as described in Shabab et al. (2014)] and D_5_-/D_6_-JA all at the same concentration.

### Extraction and Quantification of GABA

For GABA measurements amino acids were extracted with methanol following the single leaf extraction protocol for phytohormones (Jimenez-Aleman et al., 2015). The methanolic extract was diluted 1:10 (v/v) with water containing 10 μg/ml of ^13^C, ^15^N algal amino acid mix (Isotec, Miamisburg, OH, United States) as internal standard. GABA was determined using LC-MS/MS according to Scholz et al. (2015).

### Quantitative Real Time (qRT)-PCR

RNA extraction, DNase treatment, reverse transcription and qRT-PCR were carried out as described in Heyer et al. (2018). The expression level of *JAZ10* was quantified with the 2^-ΔCT^ method (Livak and Schmittgen, 2001; Ohlen et al., 2016) using *RPS18B* as housekeeping gene, were 2^-ΔCT^ = 2^CT^_RPS18_/2^CT^_JAZ10_. Untreated plants were used as controls. Primers used for *RPS18B* and *JAZ10* have been reported in Scholz et al. (2014).

### Statistics

All statistical analysis was performed using RStudio Version 1.0.143. Statistic significant differences were tested using one sample or two sample Wilcoxon Test as indicated in the figure legends. False discovery rate (FDR) (Benjamini and Hochberg, 1995) was applied on all tests. For Phytohormone measurements a total number of biological replicates of at least *n* = 11 was used. In case of *JAZ10* expression studies the number of biological replicates was at least *n* = 5 and in case of GABA quantification it was at least *n* = 6. For detailed information of the number of biological replicates (*n*) used in each experiment, see the figure legends.

## Results

### Sampling Induces Systemic Jasmonate Signaling

In order to investigate the systemic pattern of phytohormones in the *Arabidopsis* rosette, plants were wounded on leaf number 8 and all leaves from 5 to 13 were collected. The mechanical larva MecWorm (Mithöfer et al., 2005) was used for wounding treatment, since in comparison to previous studies (Glauser et al., 2008a, 2009; Chauvin et al., 2013) a constantly wounding of the leaf should be achieved to mimic the mechanical damage of real insect feeding. The midrib of the leaves was included into wounding to ensure a systemic wound response (Kiep et al., 2015). Phytohormones were measured in each leaf after 1 h of treatment. The time point was chosen according to previous investigations on systemic jasmonate related gene expression (Mousavi et al., 2013). Leaves were collected starting from oldest (leaf 5) to the youngest (leaf 13). Phytohormone contents of the individual leaves are displayed in Figure 1.

Jasmonates are known to be main mediators in wound signaling in plants (Koo and Howe, 2009) and there were hints that they accumulate in systemic leaves (Glauser et al., 2009; Koo et al., 2009; Chauvin et al., 2013; Jimenez-Aleman et al., 2015). As expected we saw a strong local response in case of OPDA, JA and JA-Ile. All three jasmonates were significantly induced upon wounding in leaf 8 (Figure 1). However, compared to leaves of non-treated plants just for JA a small but significant increase in two systemic leaves of treated plants was measured, in the indirectly connected leaves 5 and 6 (Figure 1B). In contrast to previously published results, no difference could be shown in the directly connected leaf 13 (Glauser et al., 2009; Chauvin et al., 2013).

Furthermore, the jasmonate JA and JA-Ile content in younger leaves was detected to be much higher, even in untreated leaves, than in older leaves (Figure 1). In order to test if this rise in jasmonate content was age dependent or a matter of the sampling direction, we collected leaves from untreated plants from old to young leaves and the other way around. Independent of the sampling direction the level of OPDA in younger leaves was higher than in older leaves (Figures 2A,B). Strikingly, for JA as well as JA-Ile, the sampling direction was detected to cause differences in the phytohormone level between younger and older leaves. If leaves were collected from young to old, JA and JA-Ile levels increased toward the older leaves (Figures 2D,F); if collected from old to young they increased toward younger leaves (Figures 2C,E). This shows that cutting the leaves to sample plant material is already sufficient to induce systemic JA and JA-Ile accumulation.

**FIGURE 3:**
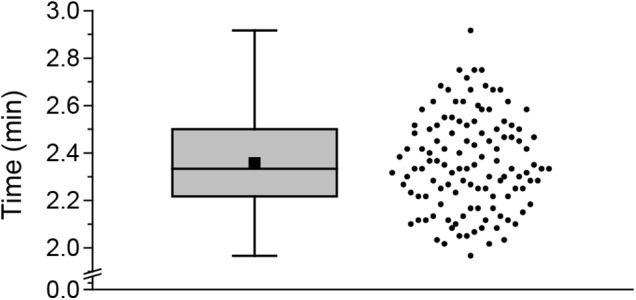
Sampling time for one *Arabidopsis* rosette. Boxplot (left) and scatter plot (right) of times needed for sampling 9 leaves. Both plots show the distribution of the measured data. The scatter plot shows each time measured. The box indicates the middle 50% of the data points. The black square indicates the mean value and the line within the box the median. Whiskers are defined as 1.5 fold interquartile range. In total, time was measured for 119 sampling events.

To gain further insight into the speed of the cutting-induced systemic JA and JA-Ile elevation, we measured the time that is necessary to harvest all nine leaves of interest. The average sampling time was 2.36 min (Figure 3). Since sampling-induced JA and JA-Ile elevation started after cutting 3 to 5 leaves, it took less than 1.57 min to induce these phytohormones systemically.

**FIGURE 4:**
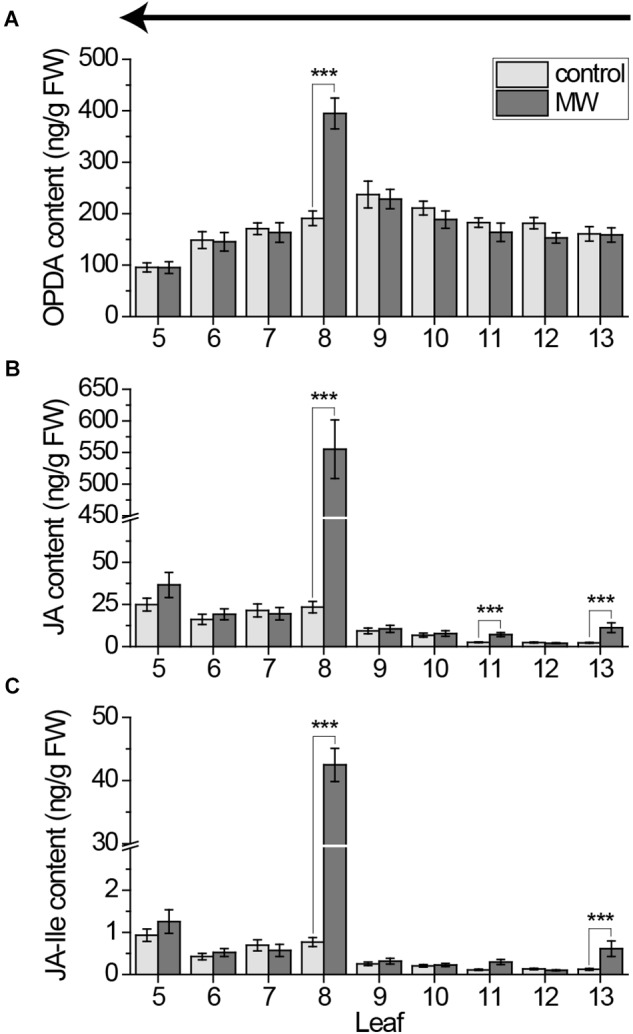
Jasmonate content in individual *Arabidopsis* leaves after wounding, sampled with the cut-and-freeze-method from young to old leaves. Mean levels of OPDA **(A)**, JA **(B)**, and JA-Ile **(C)** ±SE in individual leaves after wounding leaf 8 with MecWorm for 1 h. Untreated plants were used as control. Legend for color code see **(A)**. Leaves were collected with the cut-and-freeze-method from leaf 13 (young) to leaf 5 (old) as indicated by the arrow in **(A)**. The experiment was repeated three times independently (*n* ≥ 13). Statistically significant differences between phytohormone content of treated and untreated plants within the leaves were determined by unpaired two-sample Wilcoxon test. Asterisk indicates significance (^∗∗∗^*P* < 0.001). *P*-values are FDR corrected.

To investigate whether these sampling-induced signals are as high as the wounding treatment-induced signals and thus causing false negative results, we repeated the 1 h MecWorm treatment with collecting leaves from young to old and measured the jasmonate content. In line with the data from the untreated plants (Figures 2A,B), OPDA was only locally but not systemically induced, providing evidence that OPDA is not fast induced (Figure 4A). In contrast, JA and JA-Ile levels strongly increased in the wounded leaf 8 as well as in the directly connected leaf 13 and, in case of JA, in the indirectly connected leaf 11 (Figures 4B,C). However, the significant induction in leaves 5 and 6 found after harvesting leaves from 5 to 13 (Figure 1) was not visible anymore. Thus, the systemic JA und JA-Ile elevation might follow the vascular connections of the leaves, but sampling-induced signals attenuate the treatment effects and impede a reliable analysis of the whole rosette pattern at once.

**FIGURE 5:**
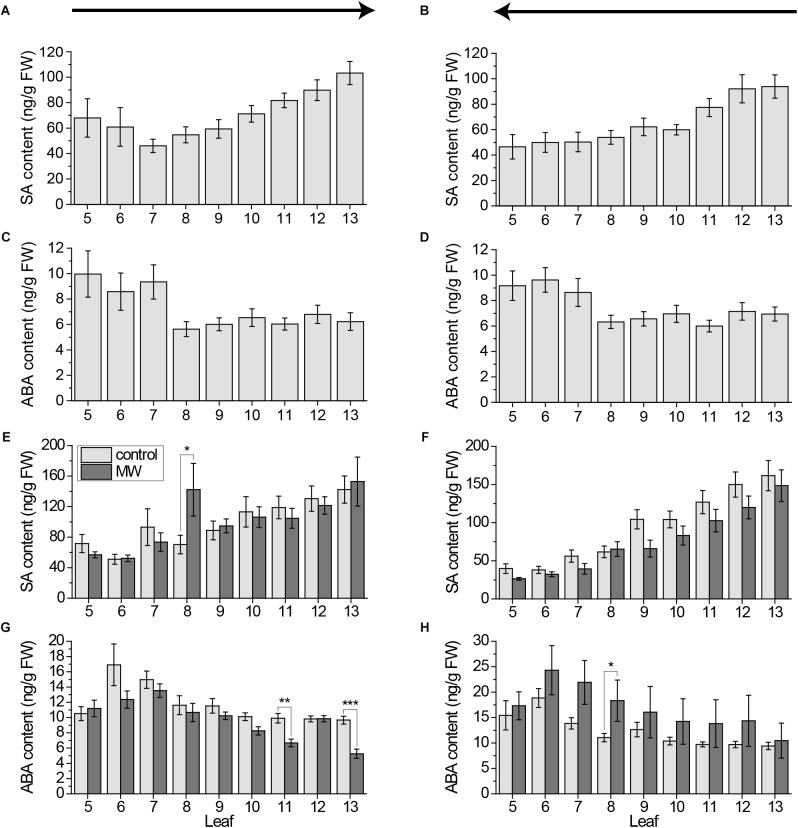
Systemic pattern of SA and ABA in individual leaves of the *Arabidopsis* rosette. Mean levels of SA **(A,B,E,F)** and ABA **(C,D,G,H)** ±SE in individual leaves of *Arabidopsis* plants. In **(A–D)** untreated plants were used. Experiment was repeated two times independently (*n* ≥ 11). In **(E–H)** plants were wounded for 1 h with MecWorm and phytohormone content of the leaves was compared to unwounded plants. See **(E)** for color coding legend. Experiment was repeated at least three times independently (*n* ≥ 13). All samples over all experiments were collected using the cut-and-freeze-method. Sampling direction is indicated by arrows above the figures. In **(A,C,E)** and **(G)** rosette was collected from old (5) to young leaves (13) and in **(B,D,F,H)** from young to old leaves. Statistically significant differences between phytohormone content of treated and untreated plants within the leaves in experiments **(E–H)** were determined by unpaired two-sample Wilcoxon test. Asterisk indicates significance (^∗^*P* < 0.05, ^∗∗^*P* < 0.01, and ^∗∗∗^*P* < 0.001). *P*-values are FDR corrected.

Because of the known crosstalk between Jasmonates and SA as well as ABA (Koornneef and Pieterse, 2008; Vos et al., 2013), we next investigated the systemic pattern of these hormones. To proof if the sampling direction has any effect on the levels of these two phytohormones, we first measured the SA and ABA level in untreated plants, when sampled from old to young and from young to old. Both SA and ABA were not influenced by the sampling direction (Figures 5A–D). The SA level is decreasing with the increasing leaf age, while ABA is increasing slightly independent on the sampling direction. We further investigated if these hormones play a role in systemic signaling after wounding. Neither SA nor ABA showed a clear systemic pattern after 1 h MecWorm treatment, independent on the sampling direction (Figures 5E–H). Just sporadically few leaves showed a significant increase or decrease of either SA or ABA, which might be rather artifacts than real systemic signals.

### *JAZ10* Expression Is Unaffected by Sampling

**FIGURE 6:**
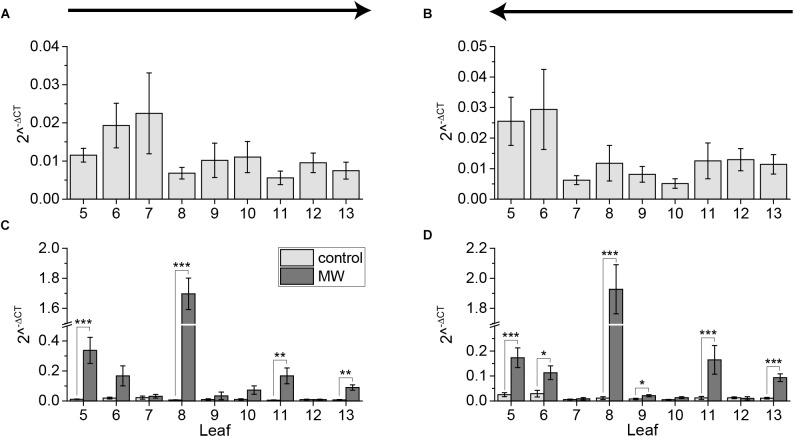
Systemic pattern of *JAZ10* gene expression. Mean ± SE expression of *JAZ10* gene in untreated control plants **(A,B)** and in comparison to plants treated for 1 h with MecWorm at leaf 8 **(C,D)**. For color coding see legend in **(C)**. Individual leaves were collected using the cut-and-freeze-method. Sampling direction is indicated by arrows above the figures. In **(A)** and **(C)** rosette was collected from old (5) to young leaves (13) and in **(B)** and **(D)** from young to old leaves. Statistically significant differences between *JAZ10* expression levels of treated and untreated plants within the leaves in experiments **(C)** and **(D)** were determined by unpaired two-sample Wilcoxon test. Asterisk indicates significance (^∗^*P* < 0.05, ^∗∗^*P* < 0.01, and ^∗∗∗^*P* < 0.001). *P*-values are FDR corrected. Whole experiment was repeated at least two times independently (*n* ≥ 5).

Previous studies showed that the jasmonate-regulated gene *JAZ10* is upregulated in systemic leaves that are vascularly connected to the wounded leaf, but not in non-connected leaves (Glauser et al., 2009; Mousavi et al., 2013). Because we showed that sampling induces JA and JA-Ile to the same extend as 1 h wounding by MecWorm, we examined if sampling with the cut-and-freeze-method can induce jasmonate-regulated gene expression as well. Therefore, plants were 1 h MecWorm-treated and *JAZ10* expression was determined using qRT-PCR, sampling once from young to old and from old to young leaves. The sampling direction had no effect on the systemic *JAZ10* expression. The expression levels in leaves of untreated plants were comparable between the two sampling directions (Figures 6A,B). However, after wounding leaf 8, the *JAZ10* expression increased in the local leaf as well as in the directly connected leaf 13 and the indirectly connected leaves 5 and 11 (Figures 6C,D) in both sampling directions. Leaf 6 showed an increase in *JAZ10* expression over both treatments as well, even though this result was just significant when sampling from young to old leaves (Figures 6C,D). However, this leaf is known as a variable leaf (Mousavi et al., 2013). Surprisingly, the unconnected leaf 9 displayed a small but significant difference between control and treatment when sampling from young to old (Figure 6D). Nevertheless, this increase in *JAZ10* expression was quite low compared to the increase in other systemically connected leaves and thus might be not of biological relevance. Taken these results together, in contrast to the jasmonate levels the expression of the jasmonate regulated gene *JAZ10* was not induced by sampling.

### Freeze-and-Cut-Method Overcomes Sampling Induced Signaling

**FIGURE 7:**
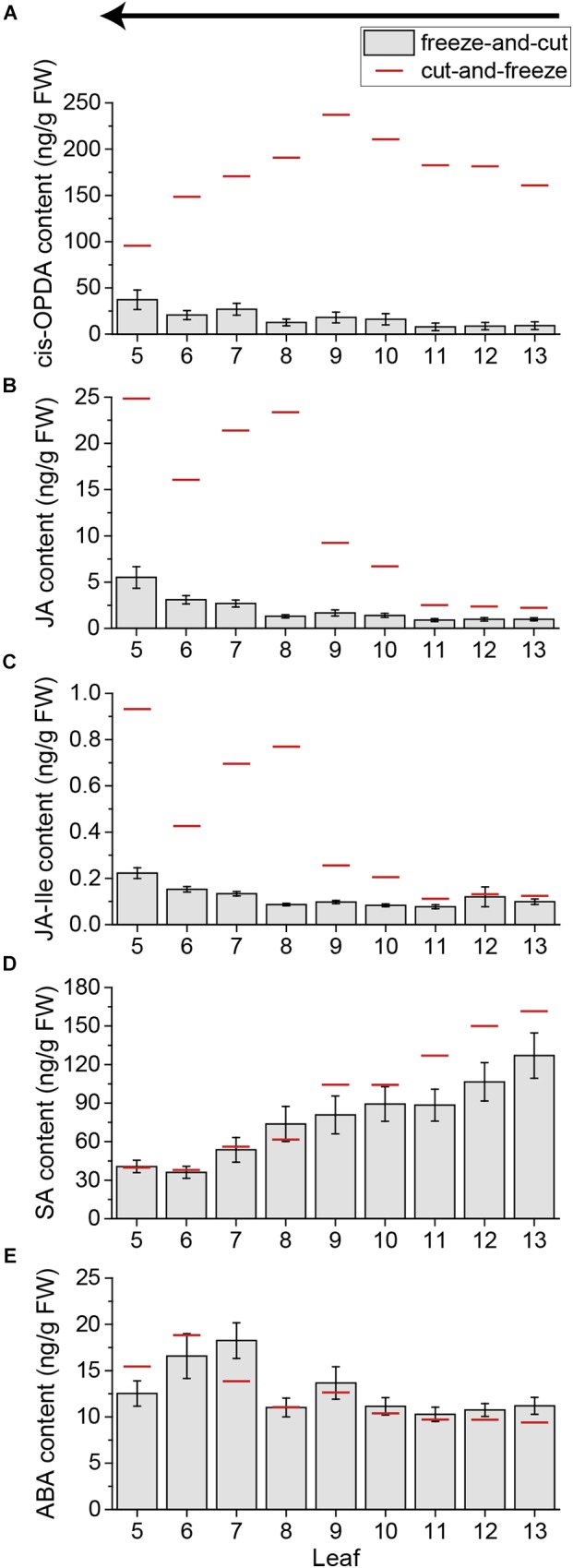
Phytohormone content of untreated *Arabidopsis* leaves using the freeze-and-cut-method. Bars represent mean levels of OPDA **(A)**, JA **(B)**, JA-Ile **(C)**, SA **(D)**, and ABA **(E)** ± SE in individual leaves of untreated *Arabidopsis* plants. Individual leaves were collected with the freeze-and-cut-method from leaf 13 (young) to leaf 5 (old). Sampling direction is indicated by an arrow above the figures. The experiment was repeated six times independently (*n* ≥ 19). Red lines indicate the mean values of untreated plants collected with the cut-and-freeze-method in the same sampling direction (see Figures 4, 5F,H).

Because of the sampling time needed for a whole *Arabidopsis* rosette, the cut-and-freeze-method was no longer appropriate as shown above. To overcome the problem of sampling-induced phytohormone elevations, a new sampling method was developed. It was shown before that rapid freezing of the plant material can decrease the amount of sampling induced JA and JA-Ile (Glauser et al., 2008b). Thus, we tested if freezing the sample before cutting (freeze-and-cut-method) might solve the problem of sampling-induced phytohormone elevations. We froze untreated plants by dipping the whole rosette in liquid nitrogen and cut the frozen leaves from young to old ones, to avoid destruction of the leaves. The new freeze-and-cut-method drastically decreased the levels of OPDA, JA, and JA-Ile in all leaves (Figures 7A–C). Thereby the sampling-induced increase of JA and JA-Ile in older leaves was reduced as well; the amount of JA could be lowered up to 18 times and the amount of JA-Ile up to 9 times. (Figures 7B,C). In line with the results above, SA and ABA levels were not influenced by the sampling method. The amount of SA and ABA in single leaves of the freeze-and-cut-method was comparable to those of the cut-and-freeze-method (Figures 7D,E). Thus the freeze-and-cut-method can be used to investigate systemic phytohormone patterns by sampling the whole rosette of *Arabidopsis*.

### JA and JA-Ile Are the Key Systemic Phytohormones Mediating Early Wound Responses

**FIGURE 8:**
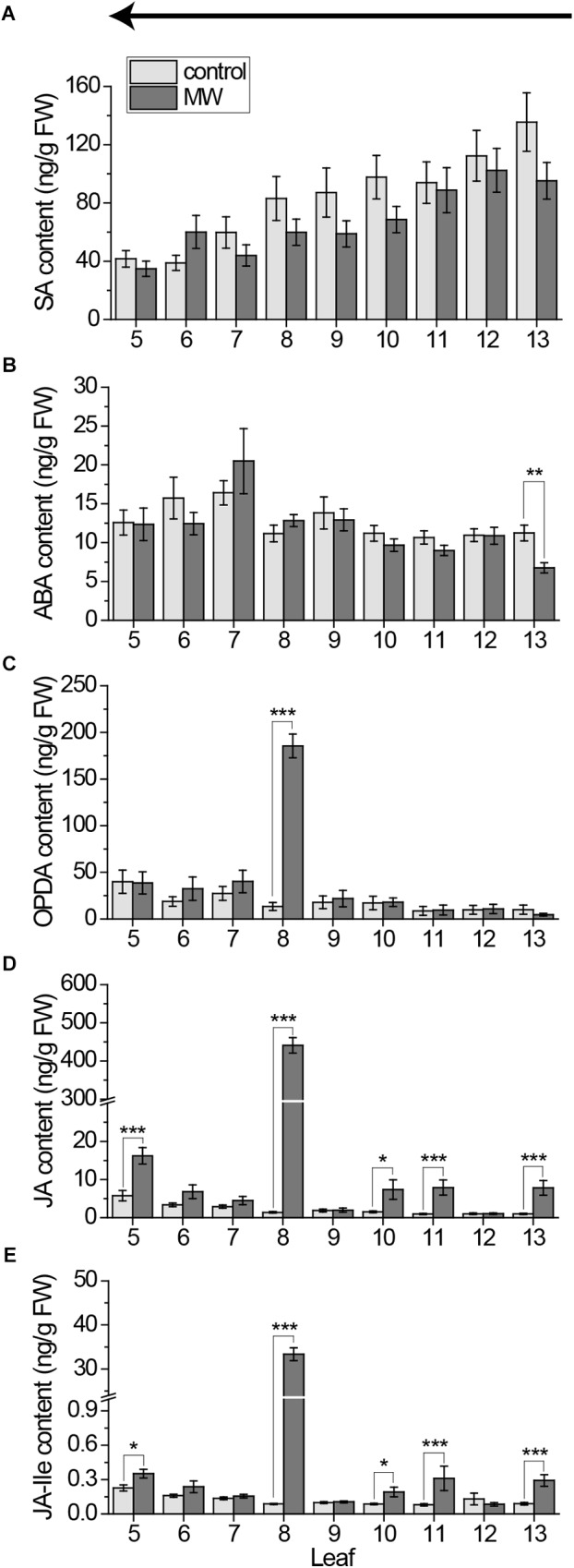
Systemic pattern of phytohormones in individual leaves of the *Arabidopsis* rosette after wounding using the freeze-and-cut-method for sampling. Mean levels of SA **(A)**, ABA **(B)**, OPDA **(C)**, JA **(D)**, and JA-Ile **(E)** ± SE in individual leaves after wounding leaf 8 with MecWorm for 1 h.*(Untreated plants were used as control. Legend for color code see **(A)**. Leaves were collected with the freeze-and-cut-method from leaf 13 (young) to leaf 5 (old) as indicated by the arrow above the figures. The experiment was repeated five times independently (*n* ≥ 16). Statistically significant differences between phytohormone content of treated and untreated plants within the leaves were determined by unpaired two-sample Wilcoxon test. Asterisk indicates significance (^∗^*P* < 0.05, ^∗∗^*P* < 0.01, and ^∗∗∗^*P* < 0.001). *P*-values are FDR corrected.)*

Using the new method, we re-investigated the systemic phytohormone pattern after 1 h continuous wounding of leaf 8 with the MecWorm. The results are shown in Figure 8. In line with the results shown above, SA was neither upregulated in the local leaf nor in the systemic leaves (Figure 8A); thus, SA can be excluded as phytohormone mediating early wound responses both locally and systemically. Also ABA seems not to be a key phytohormone in the early systemic wound response. Although a systemic reduction of ABA after wounding leaf 8 in the systemically connected leaf 13 could be measured, none of the other leaves displayed any change in the ABA level after treatment (Figure 8B). As in all measurements done before, OPDA was just upregulated locally but not systemically (Figure 8C), as reported before (Glauser et al., 2009). Only JA and JA-Ile increased locally and systemically (Figures 8D,E). Both phytohormones were upregulated in the connected leaves 13, 11, and 5. Interestingly, leaf 10 that is directly connected to leaf 5 and indirectly to leaf 13 displayed JA and JA-Ile elevation as well, suggesting a vascular connections of third order.

### Systemic GABA Elevations Are Not Influenced by the Sampling Method

**FIGURE 9:**
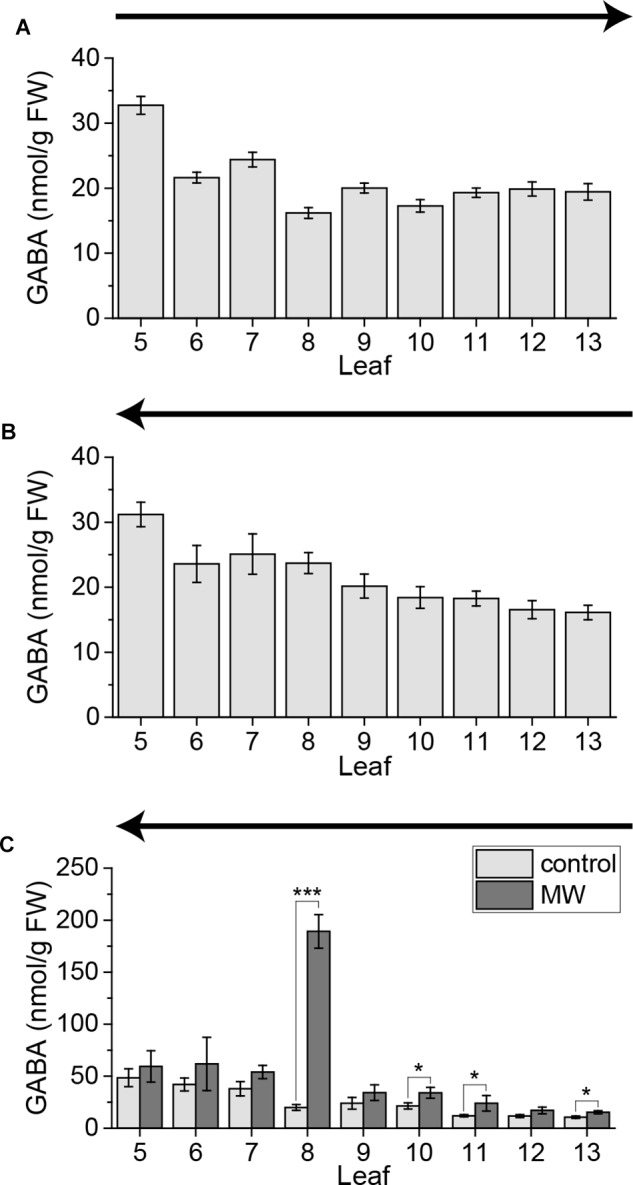
GABA content of untreated and wounded *Arabidopsis* leaves. GABA levels of individual leaves of untreated *Arabidopsis* rosettes sampled with the cut-and-freeze-method **(A,B)**. **(C)** GABA levels of individual leaves of untreated control plants and plants that were treated for 1 h with MecWorm sampled with the freeze-and-cut-method. Sampling direction is indicated in each figure by an arrow. The experiment was repeated two times independently (*n* ≥ 6) for **(A,B)** and five times independently (*n* ≥ 15) for **(C)**. Statistically significant differences in **(C)** between GABA content of treated and untreated plants within the leaves were determined by unpaired two-sample Wilcoxon test. Asterisk indicates significance (^∗^*P* < 0.05, ^∗∗∗^*P* < 0.001). *P*-values are FDR corrected.

In previous studies it was shown that the non-proteinogenic amino acid GABA is systemically induced in vascularly connected leaves upon MecWorm treatment (Scholz et al., 2015, 2017). Furthermore it was known that the local GABA level can be already upregulated within several minutes by touch (Ramputh and Bown, 1996; Bown et al., 2002) and is thus a highly sensitive wound response in plants. Therefore, we investigated if systemic GABA elevations are rapidly inducible by cutting and subsequent sampling similar to jasmonates. In order to test this, we cut the leaves of untreated plants from old to young and from young to old. Independent on the sampling direction the GABA level increased slightly toward the older leaves (Figures 9A,B). Thus GABA seems to be not induced by sampling.

Furthermore we measured the GABA levels in control and MecWorm-treated plants, when sampled with the freeze-and-cut-method. Similar to previous reported results (Scholz et al., 2017), the GABA level was drastically induced in the treated leaf 8 and upregulated in the directly connected leaf 13 (Figure 9C). Additionally, the indirectly connected leaf 11 and leaf 10, which likely is connected to leaf 8 *via* third order, showed a significant increase in the GABA level (Figure 9C). Nevertheless, we measured no differences in GABA concentration between control plants and treated plants in the indirectly connected leaf 5. Since induced systemic GABA levels are quite low, this might be that due to the higher variability of the GABA levels in leaf 5 (Figure 9C).

## Discussion

Phytohormones are known to play an important role as systemic signaling components after herbivory (Howe and Jander, 2008; Vos et al., 2013). For other systemic signals it is known that their distribution among the *Arabidopsis* rosette is dependent on the vascular connections between the single leaves. Up to now, most of the systemic phytohormone measurements done, did not take these connections into account. Systemic leaves are chosen randomly (Glauser et al., 2008a; Koo et al., 2009) or just few leaves that share a connection to the treated leaf were investigated (Glauser et al., 2009; Chauvin et al., 2013; Jimenez-Aleman et al., 2015). A holistic approach to measure phytohormones in the whole rosette to get insights into their distribution pattern, as done for systemic electric signals or calcium waves (Mousavi et al., 2013; Kiep et al., 2015), was missing. Furthermore, most of the studies performed, focused only on the distribution of JA and its active conjugate JA-Ile and very often jasmonate reporters are used to determine the systemic pattern of these jasmonates instead of measuring directly the phytohormone content of individual leaves (Glauser et al., 2008a, 2009; Chauvin et al., 2013; Mousavi et al., 2013; Salvador-Recatalà et al., 2014). Thus, in this study the systemic distribution of OPDA, JA, JA-Ile, SA, and ABA by measuring single leaf phytohormone contents were investigated and linked to the vascular connections between the leaves in the *Arabidopsis* rosette.

### Sampling Matters – The Cut-and-Freeze-Method Is Not Appropriate for Holistic Approaches

Glauser et al. (2008a) showed that cutting a single leaf can induce a local JA elevation within an average time of 1.2 min. To avoid such rapid local sampling-induced signals, they developed the cut-and-freeze-method (Glauser et al., 2008a,b). Nevertheless, when rosettes (leaves 5 to 13) were systematically sampled for phytohormone measurements using the cut-and-freeze-method (Figures 1–4) sampling-induced systemic JA and JA-Ile elevations were detected. Depending on the sampling direction, the amount of jasmonates increased toward the younger leaves (Figures 1B,C, 2C,E) or the older ones (Figures 2D,F, 4B,C). The systemic JA and JA-Ile increase occurred after sampling one third to one half of the rosette. Since sampling of a whole rosette takes 2.36 min (Figure 3), systemic signals induced by cutting took less than 1.57 min. These findings are supported by Chauvin et al. (2013) who reported a time of 1.5 min for the systemic JA signals to be measured in the connected leaf 13 after wounding up to 50% of leaf 8. For JA-Ile we could show that its systemic induction in leaf 13 seems to be even faster than the reported 3 min after wounding (Chauvin et al., 2013).

Interestingly, simply cutting the leaves increased the amount of systemic JA and JA-Ile to the same extend as the continuous wounding for 1 h on the local leaf 8, (Figures 1B,C, 4B,C), causing false-negative results if the cut-and-freeze-method is used for sampling the whole rosette. These data suggest that the systemic JA and JA-Ile signals are an on-off-system and independent on time of wounding at the local leaf. Once the systemic leaf is activated through its vascular connection to the local leaf, the systemic JA and JA-Ile signals are switched on. Thus, cutting one leaf after the other using the cut-and-freeze-method causes a systemic activation in the leaves that are still attached to the rosette and connected *via* the vascular system with the cut leafs. This gains high, unrealistic background levels of JA and JA-Ile already in the controls. Subsequently, if compared to these controls, the interpretation of treatment effects are affected because existing differences can be diminished. Consequently, previous results on systemic phytohormones achieved by the cut-and-freeze-method should be interpreted carefully, especially, if more than 3–5 leaves are collected consecutively.

Fast sampling-induced phytohormone elevations were detected for JA and JA-Ile only, whereas their precursor OPDA, as well as SA and ABA were not influenced by cutting the leaves (Figures 1A, 2A,B, 4A, 5). In addition, the cut-and-freeze-method seems to be just problematic, when real phytohormone levels are measured. If the *JAZ10* gene was employed for measuring systemic jasmonate responses, the controls did not display any sampling dependent increase in *JAZ10* expression (Figures 6A,B). The systemic pattern of *JAZ10* expression after wounding was independent on the sampling direction as well. The directly connected leaf 13 and the indirectly connected leaves 5 and 11 and sometimes leaf 6 showed a clear increase in *JAZ10* expression upon wounding leaf 8 (Figures 6C,D) as reported before (Mousavi et al., 2013). The differences in the outcome of the marker gene study and real jasmonate measurements using the same sampling method might be due to the time delay between phytohormone burst and the gene expression following the burst. *JAZ10* has been shown to accumulate earliest 1 h after wounding treatment (Chung et al., 2008). Sampling-induced JA and JA-Ile signals in the control plants just lasted less than 2 min before freezing the leaves. This short time period is not sufficient to transduce the signal into *JAZ10* gene expression. Hence, the fast cutting-induced JA and JA-Ile signals in the control plants are not visible, when the marker gene is taken as readout.

### JA and JA-Ile Are Key Mediators of the Early Systemic Wound Response

To avoid sampling-induced jasmonate signals, the freeze-and-cut-method was developed in this study. We showed that using this new method, the amount of jasmonates in control plants is much lower compared to those sampled with the cut-and-freeze-method (Figures 7A–C), allowing the investigation of the real systemic pattern of the phytohormones.

In line with our *JAZ10* expression data (Figure 6) and those published before (Mousavi et al., 2013), we could show that the directly connected leaf 13, and the indirectly connected leaves 5 and 11 display a significant increase in JA and JA-Ile level after treatment of leaf 8 (Figures 8D,E). In addition, a significant JA and JA-Ile increase was measured in leaf 10. It is connected to leaf 8 in third order *via* connected leaves of second and first order. Recently, Salvador-Recatalà et al. (2014) reported that if larvae cut the midvein of leaf 8 while feeding, leaf 10 is depolarized and the expression of the marker gene *JAZ10* is upregulated in leaf 10. Thus it might be possible, due to the continuous wounding along the midvein of leaf 8 in our experiment, that the systemic signal gets extended to leaves of third order. By this the radius of defended leaves gets increased (Supplementary Figure S1). However in our experiments, as well as in the experiments done by Mousavi et al. (2013), *JAZ10* expression was not increased in leaf 10 (Figures 6C,D), even though the midvein was wounded in both. It might be that such signals of third order occur with delay after those of first and second orders. Such time delay would explain why third order responses can be missed; or in case of leaf 6 are variable in *JAZ10* marker studies. However, this hypothesis needs to be proven in further studies.

Besides JA and JA-Ile, we investigated the systemic pattern of their precursor OPDA. The systemic role of this phytohormone is highly controversial. Koo et al. (2009) reported a rapid dropdown in OPDA levels 5 min after wounding in systemic leaves, used as a proof that OPDA is used as a store for fast synthesis of JA for rapid wound responses Glauser et al. (2009). measured an increase in OPDA in leaves distal to the wounded leaf about 3 min after treatment, suggesting that it is *de novo* synthesized fast after wounding. On the other hand a systemic role of OPDA after herbivore treatment was excluded by Vos et al. (2013). Here we could show, that systemic OPDA levels are not changing after continuous wounding of leaf 8 (Figure 8C) for 1 h. Furthermore, systemic OPDA levels were neither fast induced nor reduced when leaves were harvested using the cut-and-freeze-method (Figures 1A, 2A,B, 4A). Over all treatments, OPDA was just increased in local leaves (Figures 1A, 4A, 8C), like reported before after herbivory (Vos et al., 2013). This might be a hint that systemic JA and JA-Ile elevations are not due to *de novo* synthesis, but rather due to transport between the leaves as shown in *Nicotiana* plants (Sato et al., 2009, 2011). Nevertheless, it might be possible as well that they are synthesized out of the bound OPDA in the Arabidopside pool or other sources like OPCs as suggested by Jimenez-Aleman et al. (2015) and Gasperini et al. (2015). In any case, our data exclude free OPDA as rapid systemic wound signal. The same holds true for SA and ABA. None of which showed a constant local or systemic pattern over all treatments (Figures 5E–H, 8A,B). Nevertheless, SA, ABA, and OPDA might still play a role at later time points or after priming as shown for ABA (Vos et al., 2013). Taken together our results suggest that mainly JA and JA-Ile mediate the rapid systemic wound response following the vascular connections between the local and systemic leaves.

### Systemic GABA Elevations Are Not Rapid Induced by Sampling

Bown et al. (2002) reported that GABA can be locally induced by insects crawling on leaf surfaces of different plant species. First significant GABA inductions were reported after 5 min of insect crawling, but a slight trend was already seen after 2 min in tobacco leaves (Bown et al., 2002). In a previous work they reported local GABA elevations in soybeans after mechanical stimulation already after 1 min (Ramputh and Bown, 1996). We showed recently that GABA can be induced systemically after continuous wounding with MecWorm in parastichious connected leaves (Scholz et al., 2015, 2017). Here we show that simple cutting was not sufficient to trigger systemic GABA elevations (Figures 9A,B) in contrast to JA and JA-Ile elevations. It is known that systemic GABA production is independent on jasmonate signaling (Scholz et al., 2015, 2017). Therefore, it might be that systemic GABA elevations are regulated in a different time scale compared with JA and JA-Ile. Furthermore, if local GABA responses take place at first after 1–5 min (Ramputh and Bown, 1996; Bown et al., 2002), it is conceivable that sampling the whole rosette is faster than the induction of systemic synthesis of GABA. On the other hand GABA is known as a defense compound against insect herbivores (Ramputh and Bown, 1996; Scholz et al., 2015). So, it might be necessary to wound the local leaf severe and continuously like done with MecWorm, to trigger systemic GABA synthesis. Thus, we measured GABA in the whole rosette using the freeze-and-cut-method after MecWorm treatment. As reported before, the connected leaves 13 and 11 displayed a significant increase in GABA, after treatment of leaf 8 (Figure 9C), confirming that the signal inducing GABA synthesis follows the parastichious connections between the leaves (Scholz et al., 2017). Interestingly, again we could measure a systemic response in leaf 10, as already shown for JA and JA-Ile (Figures 8D,E), supporting once more the hypothesis that systemic signals might be extended to third level connections.

## Conclusion

In this work a holistic approach for systemic phytohormone elevations in the *Arabidopsis* rosette after herbivore-related wounding was performed. Detailed investigation of jasmonates in individual leaves revealed intrinsic problems of methods used previously to study systemic phytohormone changes. A modified method was developed that overcomes these problems and avoids false-negative results. Employing the new method in combination with targeted analysis of different phytohormones it became clear that very likely JA and JA-Ile are the key phytohormones involved in a rapid systemic response. The systemic jasmonate response seems to be independent on changes in free OPDA levels. It was also demonstrated that these systemic signals strictly follow the vascular connections of first, second, and third orders between the leaves. The distance that can be bridged between a locally treated and systemically activated leaves might depend on the kind of wounding treatment and the nature of the yet non-identified traveling signal(s). This needs to be studied in future.

## Author Contributions

MH and AM conceived and designed the research and interpreted the data. MH and MR performed the experiments and analyzed the data. MH, AM, and MR wrote the manuscript.

## Conflict of Interest Statement

The authors declare that the research was conducted in the absence of any commercial or financial relationships that could be construed as a potential conflict of interest.
