# Supplementary material for: A Holistic Approach to Analyze Systemic Jasmonate Accumulation in Individual Leaves of Arabidopsis Rosettes Upon Wounding
Source: Front Plant Sci. 2018 Oct 30;9:1569. doi: 10.3389/fpls.2018.01569 (PMC6218591; doi:10.3389/fpls.2018.01569)
Supplement: Supplementary file 1 [file Image_1.pdf]

Supplementary Material

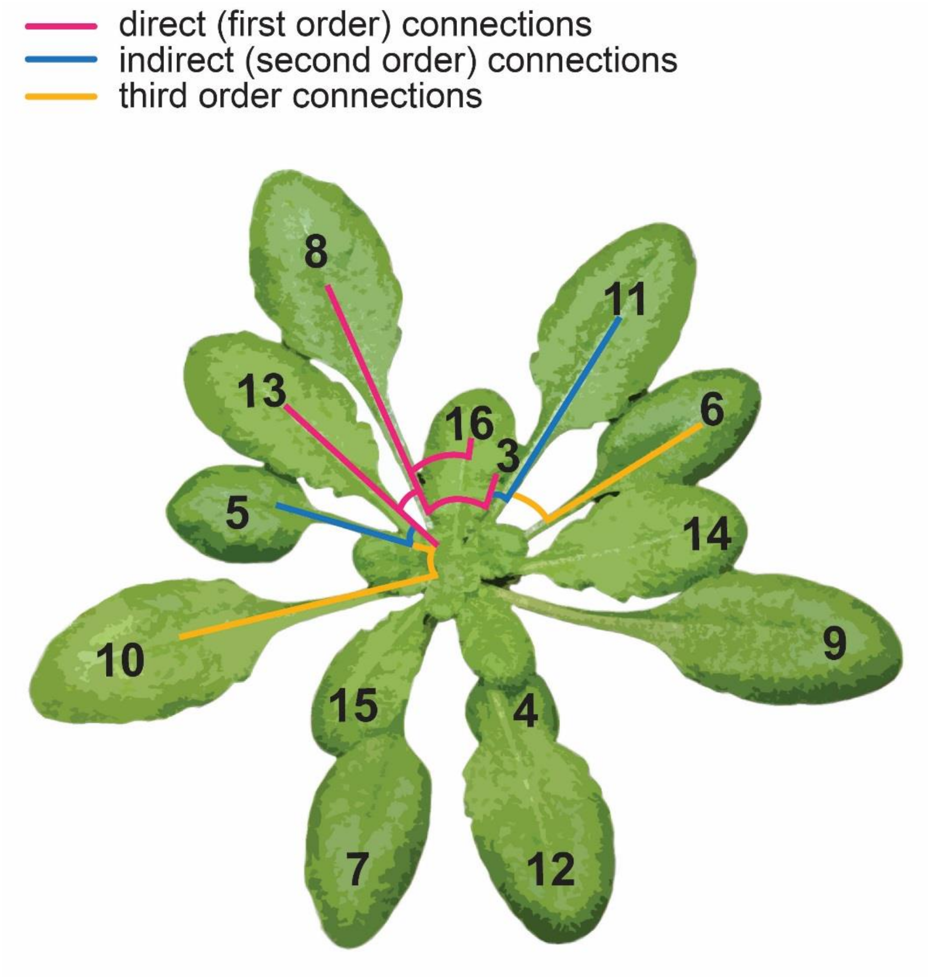

Figure S1. Vascular connections of leaf 8 in a 5-week-old *Arabidopsis thaliana*. Shown are the vascular connections of leaf 8 that was used as local leaf for all treatments. Red lines indicate first order connections (8±5 and 8+8). Those leaves share a direct connection to leaf 8. Blue lines indicate second order connections (8±3). Leaves are indirectly connected to leaf 8. Yellow lines indicate possible third order connections to leaf 8 via indirectly or directly connected leaves of leaf 8.
